# Contribution of Deep Learning in the Investigation of Possible Dual LOX-3 Inhibitors/DPPH Scavengers: The Case of Recently Synthesized Compounds

**DOI:** 10.3390/bioengineering9120800

**Published:** 2022-12-14

**Authors:** Dimitrios Bakalis, George Lambrinidis, Angeliki Kourounakis, George Manis

**Affiliations:** 1 Department of Computer Science and Engineering, School of Engineering, University of Ioannina, 45110 Ioannina, Greece; 2 Division of Pharmaceutical Chemistry, Department of Pharmacy, School of Health Sciences, National & Kapodistrian University of Athens, 15771 Athens, Greece

**Keywords:** anti-inflammatory, antioxidant, deep learning, classification, regression, LOX-3, DPPH

## Abstract

Even though non-steroidal anti-inflammatory drugs are the most effective treatment for inflammatory conditions, they have been linked to negative side effects. A promising approach to mitigating potential risks, is the development of new compounds able to combine anti-inflammatory with antioxidant activity to enhance activity and reduce toxicity. The implication of reactive oxygen species in inflammatory conditions has been extensively studied, based on the pro-inflammatory properties of generated free radicals. Drugs with dual activity (i.e., inhibiting inflammation related enzymes, e.g., LOX-3 and scavenging free radicals, e.g., DPPH) could find various therapeutic applications, such as in cardiovascular or neurodegenerating disorders. The challenge we embarked on using deep learning was the creation of appropriate classification and regression models to discriminate pharmacological activity and selectivity as well as to discover future compounds with dual activity prior to synthesis. An accurate filter algorithm was established, based on knowledge from compounds already evaluated in vitro, that can separate compounds with low, moderate or high activity. In this study, we constructed a customized highly effective one dimensional convolutional neural network (CONV1D), with accuracy scores up to 95.2%, that was able to identify dual active compounds, being LOX-3 inhibitors and DPPH scavengers, as an indication of simultaneous anti-inflammatory and antioxidant activity. Additionally, we created a highly accurate regression model that predicted the exact value of effectiveness of a set of recently synthesized compounds with anti-inflammatory activity, scoring a root mean square error value of 0.8. Eventually, we succeeded in observing the manner in which those newly synthesized compounds differentiate from each other, regarding a specific pharmacological target, using deep learning algorithms.

## 1. Introduction

The continuous need for better, more effective and safer drugs, creates new challenges for scientists. Laboratory experiments, needed to be conducted for this purpose i.e., the development of new drugs, are not only time consuming but also costly. Computer science is called upon to provide potential solutions utilizing pharmacological/pharmacochemical data analysis together with machine and deep learning to minimize efforts and costs [1,2]. The advancement of deep learning has given the opportunity to apply models to real world data and to aquire quite accurate predictions whether being stock predictions, natural language classification, time series predictions or other. These models are suited for classification problems as well as regression problems. This means that the models can be trained to the point that they can categorize different types of behavior given a set of pharmacological/pharmacochemical input or predict the exact value of effectiveness and selectivity.

Previous work showed that there is a possibility to predict the activity of newly synthesized compounds when using simple machine learning models in a simulated environment. The compounds selected are conjugates of commonly used NSAIDs (Non-steroidal anti-inflammatory drugs) (see earlier paper Tzara et al. [3]) fused with the antioxidant moieties 3,5-di-tert-butyl-4-hydroxybenzoic acid (BHB), its reduced alcohol 3,5-di-tert-butyl-4-hydroxybenzyl alcohol (BHBA), or 6-hydroxy-2,5,7,8-tetramethylchromane-2-carboxylic acid (Trolox), a hydrophilic analogue of α-tocopherol. The acidic character of the NSAID was reduced in order to ensure a safer profile for their use, especially regarding their gastrointestinal toxicity. The fusion of the used NSAIDs and their corresponding alcohols with the antioxidants BHB, BHBA and Trolox not only leaves their antioxidant profile intact, but further improves it.

In this study we utilized deep learning models, tuned for classification as well as for regression problems, to investigate whether we can create and train a neural network to the point that it can separate and recognize different types of pharmacological activity, anti-inflammatory and/or antioxidant, regarding newly synthesized compounds. We analyzed the performance and the behavior of our models using two sets of data and we made predictions on a set of test compounds that were cross-validated with experimentally derived results to evaluate the robustness of our classification and regression protocols for future compounds. We tried to create models capable of predicting the specific activities prior to synthesis. To achieve our goals we used a wide range of machine learning algorithms, utilizing both linear and non-linear models, and two different architectures regarding the deep learning models. The architecture of the models is simple enough to be run on any home office computer.

We conducted an extensive analysis of the compounds of interest from the scope of computer science and obtained results that would be otherwise difficult to obtain in the lab. Specifically, we trained advanced fine-tuned deep learning models that were not only able to classify compounds between two possible classes but also to predict the exact value of effectiveness given a single compound for a specific target. Additionally, the models were able to point out compounds that could possibly have dual biological activity, being simultaneously lipoxygenase-3 (LOX-3) inhibitors and 2-diphenyl-1-picrylhydrazyl (DPPH) scavengers.

The interest for this study was the opportunity to investigate and analyze real world pharmacological data, from the scope of bioinformatics, for potentially reliable results for the scientific community. Another goal was to study the behavior of compounds in a simulated environment in order to eliminate possible errors that could occur in the lab. The rising discipline of systems pharmacology and polypharmacology is based on such studies for more effective but less toxic therapeutic agents [4,5,6]. Additionally, with the use of deep learning, we can run a large number of tests without considering the costs for the experiments that would otherwise need to be performed in a physical lab. Furthermore, the lack of similar studies in the field of biomedical data made this work even more interesting, foreseeing very promising results and creating new paths and opportunities for future research.

## 2. Materials and Methods

### 2.1. Data

In this study we used two sets of data consisting of synthesized compounds with anti-inflammatory (LOX-3 inhibitors) and/or antioxidant activity (DPPH scavenging). We focused on those specific targets among several others, because in our laboratory we have established reliable in vitro tests for those specific targets and our test set compounds have been validated on LOX-3 and DPPH, respectively. The molecular descriptors of all compounds are calculated using the Molecular Descriptors module, as implemented on Schrodinger Suite of Software 2021, and stored as numerical features that represent different chemical properties for each and every compound. Some of the properties are Atom Count, Bond Count, Centralization, Eccentricity etc. The database from which the sets were extracted is the ChEMBL database [7].

Regarding the first data set, which is presented in Table 1, we have 2 classes that concern two different types of activity. Compounds that belong in the first category have LOX-3 inhibitory activity (as indication of anti-inflammatory activity) and compounds that belong in the second category have DPPH inhibitory activity (as an indicator of antioxidant activity). The numerical features of the compounds are 277 molecular descriptors, as resulted from our study [3]. Additionally, we used a given set of 24 compounds that were synthesized and evaluated in the lab, to predict their activity using our classification models. The annotation of the 24 test compounds is referred in a our work [3].

**Figure 1:**
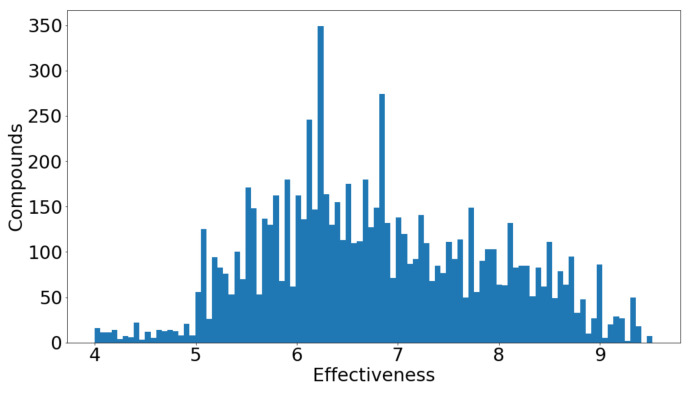
Distribution of pChEMBL values along the second data set.

The second data set, which is presented in Table 2, contains compounds that are only LOX-3 inhibitors, i.e., there is no data related to their antioxidant activity. Their activity is measured from a scale of 1 to 10, with measurements being real values. In later tables, we will be calling this activity measurement pChEMBL. Compounds that have an activity value between 1 and 3 have low effectiveness, between 4 and 7 have moderate effectiveness and between 8 and 10 have high effectiveness. This value allows a number of roughly comparable measures of half-maximal response concentration/potency/affinity to be compared on a negative logarithmic scale. For example, an $IC_{50}$ measurement of 1nM would have a pChEMBL value of 9. A histogram presenting the manner by which the compounds are distributed in the data set, depending on their value of activity, can be seen in Figure 1. The numerical features of the compounds are 277 molecular descriptors. Additionally, the annotation of the 24 test compounds is also referred to in the our work [3].

### 2.2. Data Preprocessing

In order to achieve the highest accuracy and the lowest loss for our models, we need to do the necessary preprocessing of our data. We split our data into training and validation sets in order to create an accurate classification and regression protocol that is reliable enough for our new compounds. The preparation of the first and the second data set can be seen in Table 3.

We scale the features of our compounds using the MinMaxScaler normalization method. Some machine learning models do not perform well without data normalization, so the MinMaxScaler is highly recommended with these specific data sets where we have big fluctuation in values between our features. The method can be expressed as a formula of the form:

(1) $${\hat{x}}_{i}=\frac{x_{i}-x_{min}}{x_{max}-x_{min}}$$

Additionally, we had to transform the classes of the first data set, in order for each class to be a probabilistic representation of the class that a compound belongs to. The technique that was used to transform the classes is called One-Hot Encoding and it is commonly used on multi class problems. For example, if a compound belongs to category 1, the class will be transformed into a one dimensional matrix with values (1, 0). Respectively, if a compound belongs to category 2 the class will be transformed into a one dimensional matrix with values (0, 1).

### 2.3. Regression Models

For this study, we used a wide variety of Machine Learning models, both linear and non-linear, suited for Regression problems. We selected the top 5 most-used models regarding general data. The models used are Linear Regressor [8], Gradient Boosting Regressor [9], Decision Tree Regressor [10], Random Forest Regressor [11] and Support Vector Regressor [12].

We tried to use the same parameters for all of our models, whenever possible, to have a general approach to the problems we are called to face. Regarding this approach, most of the machine learning models are trained for 100 epochs, using mini-batches of size 32, where the mean squared error is the considered loss. We tested many different parameters for each model to find the best ones that made the training of the models more efficient when using our two data sets.

### 2.4. Deep Learning Models

Furthermore, we wanted to analyse our data using the latest deep learning models and, specifically, artificial neural networks (ANN) as well as a convolutional neural network (CNN), as proposed in [13]. This models can be tuned for both classification and regression problems, changing the parameters of the networks. Firstly, we will discuss in depth the parameters that have been used for training and evaluating our ANN and CNN models. We created two ANN and two CNN models tuned for multi class classification problems as well as for regression problems.

For our ANN regression model, we opted with the use of root mean square error (RMSE) as the loss function as it is the most commonly used loss function in similar types of tasks. The loss metric can be expressed as a formula of the form:

(2) $$\mathrm{RMSE}=\sqrt{\sum_{i=1}^{n}\frac{{\left(\hat{y_{i}}-y_{i}\right)}^{2}}{n}}$$

The architecture of the model consists of 1 input layer, 3 hidden layers and 1 output layer. The input layer has 256 nodes and it takes as an input the features of a single compound in a form of a one dimensional matrix with a of size 283 as the number of the molecular descriptors. The activation function of the input layer is the tanh activation function [14] and can be expressed as a formula of the form:

(3) $$f\left(x\right)=tanh\left(x\right)=\frac{2}{1+e^{-2x}}-1$$

The next 3 hidden layers have 128, 64 and 32 nodes respectively and use the tanh as the activation function. Lastly, the output layer has only 1 node due to the fact that we want our model to be able to predict a decimal real value. The activation function for the output layer is the linear one, also known as the identity function.

Regarding the ANN classification model, we wanted to predict any possible dual activity that the test compounds may have. For this reason we used the categorical cross-entropy as the loss metric instead of the binary cross-entropy because we do not want deterministic results. The loss metric can be expressed as a formula of the form:

(4) $$L_{cross-entropy}\left(\hat{y},y\right)=-\sum_{i}y_{i}log\left({\hat{y}}_{i}\right)$$

The architecture of the model consists of 1 input layer, 3 hidden layers and 1 output layer. The input layer has 128 nodes and it takes as an input the features of a single compound in the form of a one dimensional matrix with size 277, as the number of the molecular descriptors. The activation function of the input layer is the tanh activation function. The next 3 hidden layers have 128, 64 and 32 nodes, respectively, and each layer is followed by a dropout layer with a value of 0.2. The activation function for the hidden layers is the tanh activation function. Lastly, the output layer has 2 nodes due to the fact that we want our model to be able to predict the probability of a compound belonging to category 1 and/or 2. The activation function for the output layer is the softmax activation function [15].

Then we created and trained a one dimensional convolutional neural network (CONV1D) classification model, again using the categorical crossentropy, as was used in the ANN classification model. A CONV1D model is much more complicated than an ANN because the main idea behind this model is that it uses convolutional layers instead of dense layers.The architecture of the model consists of 1 input layer, 3 hidden layers and 1 output layer. The input layer has 32 nodes and it takes as an input the features of a single compound in a form of a one dimensional matrix with size 277, as the number of the molecular descriptors. The activation function of the input layer is the ReLU activation function [16] and can be expressed as a formula of the form:

(5) f(x)=ReLU(x)=max(0,x)

The next 2 convolutional hidden layers have 64 and 128 nodes, respectively, with a kernel size of 3 and each layer being followed by a MaxPooling filter, with a pool size of 3. The activation function for the hidden layer is the ReLU activation function. After the final convolution layer we used the GlobalMaxPooling and Flatten methods to make the sub-data of the model suitable for the upcoming dense layers. The next dense hidden layer has 256 nodes and uses the Relu as the activation function. Lastly, as was used in the ANN classification model, the output layer has 2 nodes due to the fact that we want our model to be able to to predict the probability of a compound belonging to category 1 and/or 2. The activation function for the output layer is the softmax activation function.

Regarding the regression model, we used the same architecture as with the CONV1D classification model using 32, 64, and 128 nodes for the convolutional hidden layers, 50 nodes on the dense hidden layer and 1 node in the output layer because we want a real decimal value to be given as the output. For the convolutional layers as well as the fully connected layer, we used the tanh activation function and the loss metric of the model was the RMSE.

Table 4 explains some concepts that are used when creating a deep feed forward neural network. In this study, all deep learning models are trained for 100 epochs, using mini-batches of size 16 for the regression models and 64 for the classification models. Optimization is done with the use of the stochastic gradient descent [17] and a learning rate [18] of 0.001.

## 3. Results

We conducted our experiments using the two data sets that have been provided to us in a machine running an AMD Ryzen-5 six core processor with 16 GB of ram and an RX 580 graphics unit.

For the regression problem, we created 5 machine learning models. The metric to measure the performance of the models was the RMSE and the results on the validation set as well as the external 24 compound test set are presented in Table 5. We can see that, despite the fact that 3 out the 5 regressors have a lower RMSE value on the validation data than the support vector regressor (SVR), it still managed to outperform the other models having the best prediction on our test data set with a value of 0.93.

**Figure 2:**
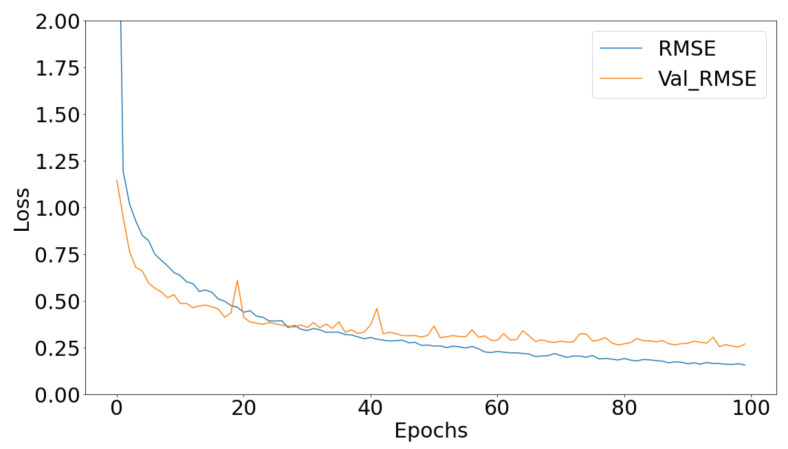
The proposed CONV1D regression protocol’s root mean square error curves.

**Figure 3:**
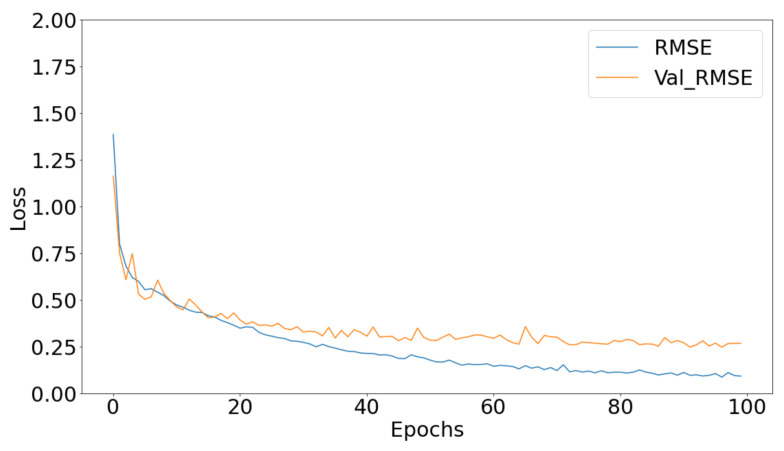
The proposed CONV1D regression protocol’s root mean square error curves.

Regarding the deep learning models, we used the ANN as well as the CONV1D regression models that were discussed in a previous section. We managed to score a loss metric of 0.28 when using the ANN and a loss metric of 0.22 when using the CONV1D, on the validation set, respectively. Both metrics for the deep models can be seen in Figure 2 and Figure 3. From the graphs we can clearly see that both models converge after 100 epochs eliminating any overfitting scenario.

Then, we predicted the activity of the test compounds using the deep regression models as well as the best scoring model from the machine learning regressors. When comparing the results to that of the pChEMBL values, the CONV1D model managed to score a RMSE value of 0.94 and a mean absolute error (MAE) value of 0.87. On the other hand, the ANN model managed to score the best results and closest to the pChEMBL values scoring an RMSE value of 0.80 and an MAE value of 0.68. The results of our predictions for the deep learning models as well as the SVR model can be seen in Table 6.

We can see that the activity range of the pChEMBL values is between 4.5 and 6. Despite not having an even distribution in our data set (Figure 1) and not having enough compounds in the range of 4 to 5 to effectively train our models, we still managed to create a strong regression protocol to predict the activity of the test compounds.

**Figure 4:**
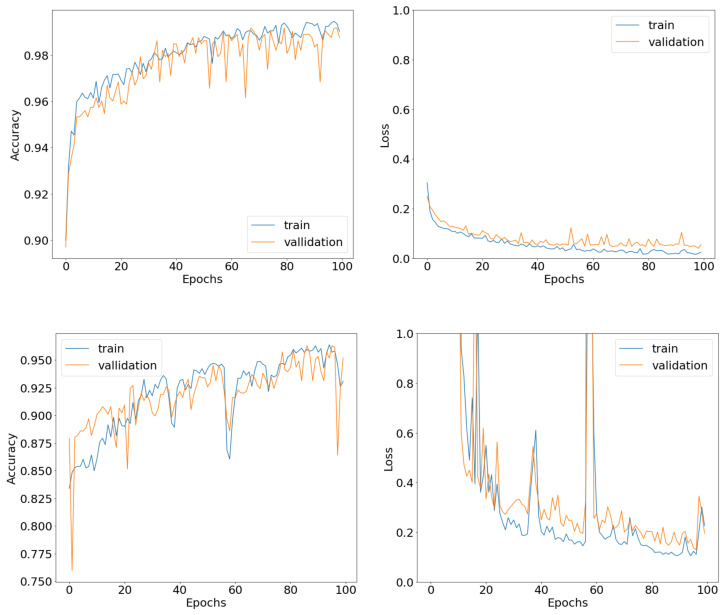
Accuracy and loss curves of the ANN as well as the CNN classification models.

Subsequently, we wanted to predict the activity of our new compounds using probabilities. With this approach we can predict any possible dual activity for a compound. We no longer treat the problem as a binary classification but rather as a categorical classification. Thus, we measured the biological activity of the newly synthesized compounds as 2 probabilities. We used the first data set to train our CONV1D classification model, achieving accuracy scores of 95.2% on the training data and 92.9% on the validation data, with the loss metric being 0.14 and 0.19, respectively. We did the same work with our ANN classification model scoring 99.8% on the training data and 99% on the validation data, with the loss metric being 0.005 and 0.049, respectively. The accuracy and loss metrics for both architectures can be seen in Figure 4.

From Figure 4 we can conclude that both models converge after 100 epochs, having almost perfect accuracy results with the loss metric brought down to a minimum. Once again, we can see that the models avoid the overfitting effect after being fine-tuned.

Further, we utilized our models to predict the activity of the test compounds and compare it with the experimental values. Previous work [3] showed that compounds 1–17 were mostly LOX-3 inhibitors (anti-inflammatory) with low dual activity. However, it is mentioned that compounds with id 9,10 and 14–17 had an indication of antioxidant activity. The indication of antioxidant activity for compounds 14–17 arises from the fact that they had DPPH scavenging $IC_{50}$ values of 34, 147, 47, and 76 µM, respectively. Additionally, compounds with ids 9 and 10 showed mild hyperlipidemic activity, which could be derived from anti-inflammatory or/and antioxidant activity. Compounds with ids 18, 20, 22, 26, 28 and 29 are the parent NSAIDs and 19, 23, 25, 27 are some reduced analogues (alcohols), utilized to synthesize compounds 1–17. Thus, our model correctly predicted, theoretically, dual activity for known anti-inflammatory drugs 18 (Ibuprofen), 20 (Naproxen), 22 (Tolfenamic acid), 26 (Mefenamic acids) 28 (Diclofenac) and 29 (Indomethacin) and moderate for their alcohol analogues.

We can tell from our predictions that, the CONV1D model manages to successfully predict the slight indications of DPPH activity, suggesting the existence of dual activity, as can be seen in Table 7.

In order to create a general protocol, we went ahead to retrain our classification models using the compounds from our first data set with the inclusion of compounds selected for two new targets, COX-2 and Nrf-2, as further indicators of anti-inflammatory and antioxidant activity, respectively. We added to the initial data set 85% of the extra compounds for testing the models and 15% of the rest for validating them.

**Figure 5:**
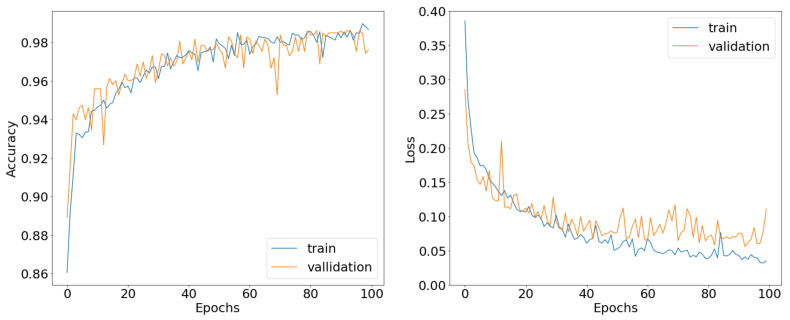

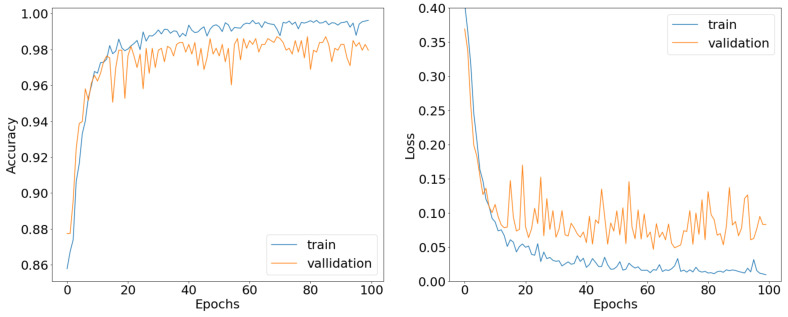
Accuracy and loss curves of the ANN as well as the CNN classification models, after the insertion of the COX-2 and Nrf-2.

The additional compounds are presented in Table 8. We used the same architectures that are discussed in the *deep learning models* subsection, for both the CONV1D and the ANN. The results that have been produced suggest that the insertion of the extra compounds even created more accurate protocols, able to better predict a more general activity (anti-inflammatory/antioxidant), regardless of the specific target involved. In particular, for the CONV1D architecture, the training accuracy improved from 95.2% to 99% and the loss metric dropped from 0.14 to 0.007. Additionally, the validation accuracy improved from 92.9% to 97.9% and the loss metric dropped from 0.19 to 0.08. The results for the accuracy and loss metrics can be seen in Figure 5. From the graphs, we can see that any overfitting scenario is eliminated when analyzing the curves of the validation sets with respect to the training curves.

## 4. Discussion

The advancement of Deep Learning over the years has opened new paths for medical research. Applications of deep learning for biomedical and pharmacological data are known to offer solutions to various problems, such as the ones that we have tackled in this study. Review papers in the field summarize research activities and trends. Some of them focus on machine learning [19,20] while others emphasize deep learning [21,22]. Categorizing and analyzing different types of compounds active on an enzyme (lipoxygenase-3) requires precise calculations and experiments to be able to produce reliable data and results when developing new compounds. In the current literature, those kinds of problems are being tackled using machine learning models such as SVR, KNN etc.

In our study, we determined the significance of utilizing a custom CONV1D model on non-image data using the categorical cross-entropy instead of the binary cross-entropy when trying to categorize newly synthesized compounds. A major finding of this study was the ability of the deep learning models, both the classification and regression models, to be able to predict whether a compound had a single or dual activity with a precise measurement outperforming the conventional machine learning models that have been used so far in the literature. The parameters as well as the architecture of our deep models showed that we can train more effective and reliable models when we wish to predict a real value regarding a regression problem. In particular, our deep regression models were able to predict the exact value of LOX-3 inhibitory activity given to new compounds and outperformed by far the conventional regression protocols, showing the power of a deep model.

The main advantage of using a CONV1D model over an ANN model or other machine learning models, when used for pharmacological activity, is that it enables us to build a hierarchy of local and sparse features derived from spectral and temporal profiles while the other models build a global transformation of features.

The deep learning protocols could be much more reliable if we have data sets with more compounds for testing as well as an even distribution of the categories in our data sets. However, we still managed to create accurate neural networks that are able to predict the test compounds after cross-validating them with the lab results. Our models can filter designed molecules prior to synthesis, regarding their potency against the targets studied. However, it is difficult to retrieve the features responsible for potency and project them to our designed molecules.

In future work, we plan on using both regression and classification protocols in designing more potent compounds prior to synthesis on the basis of a rational drug design project. Especially the latter generalized model containing two protein targets for each activity will facilitate our efforts and we intend to include even more protein targets. Finally, we shall use them for unsupervised learning purposes in a wide variety of biomedical data.

## 5. Conclusions

In this study, we proposed the application of both machine learning and deep learning models on pharmacological data and showed the superiority of deep neural networks when tackling issues related to the development of new potentially active compounds. We tested 5 different machine learning models, both linear and non-linear on our data, and showed both the ability to be trained effectively and to predict new designed compounds. We implemented two deep learning architectures and effectively trained them on our data scoring, with results quite close to the experimental ones.

We evaluated and fine-tuned the parameters of the models and created two accurate deep classification and six regression models. The models are able to categorize compounds based on their biological activity and also predict the exact value of effectiveness for specific compounds. Our experimental results suggested that deeper features always lead to more accurate classification protocols despite not having evenly distributed data sets. In conclusion, the use of a custom CONV1D model allowed us to predict the existence of dual activity compounds in a simulated environment, without utilizing lab resources, thus creating a reliable protocol for further operational use on pharmacological data prediction.

## Figures and Tables

**Table 1:** Presentation of the first data set.

Activity	No. of Compounds	Features	Class Code
LOX-3 Inhibitors	4383	277	1
DPPH Scavenging	469		2

**Table 2:** Presentation of the second data set.

Activity	No. of Compounds	Features	pChEMBL
LOX-3 Inhibitors	8182	277	1–10

**Table 3:** Data preparation.

Protocol	Target	Training Data	Validation Data	Test Data
Classification	LOX-3	3741	642	24
	DPPH	383	86	
Regression	LOX-3	6545	1637	24

**Table 4:** Methodologies used for the creation of a deep neural network with details.

Function	Details
Convolution-1D	Sliding window convolution to 1-dimensional input information.
ReLU	Performs linear rectification activation of the input vector of neural network layer and outputs nonlinear results.
MaxPooling	Selects the highest value on the spatial domain signal given an input window.
GlobalMaxPooling	Ordinary max pooling layer with the pool size being equal to the size of the input.
Flatten	Used to transform the multidimensional output of a convolutional layer to a one-dimensional array.
Dropout	Regularization layer to prevent any possible overfitting.
Dense	Most commonly used layer in machine learning. It consists of nodes that are directly connected to their preceding layer.

**Table 5:** RMSE results using the second data set.

Compound ID	Validation Set	Test Set
Support Vector	0.64	0.93
Gradient Boosting	0.59	1.06
Random Forest	0.40	1.56
Decision Tree	0.57	1.72
Linear	0.70	371.16

**Table 6:** Comparison between activity predictions and pChEMBL.

Compound ID	CONV1D	SVR	ANN	pChEMBL
1	5.46	6.00	6.70	6
3	5.73	5.98	5.92	5.52
4	5.59	6.04	6.18	5.4
5	6.06	6.05	6.38	5.3
6	5.88	5.40	4.06	5.22
9	5.97	5.94	5.18	5.05
10	6.23	6.16	6.15	5
11	6.09	6.16	6.26	4.96
12	5.80	6.48	5.91	4.92
13	6.14	6.45	5.88	4.89
14	6.14	6.04	5.92	4.85
15	6.14	6.11	5.94	4.82
16	5.92	6.02	6.13	4.8
17	5.93	5.69	4.56	4.77
18	5.22	5.01	4.46	4.74
19	5.26	4.81	4.50	4.72
20	5.53	3.94	5.23	4.7
22	5.38	4.31	4.26	4.66
23	5.14	4.55	4.44	4.64
25	5.66	5.46	4.27	4.6
26	5.58	5.92	5.76	4.59
27	5.70	5.20	4.13	4.57
28	5.23	5.84	4.44	4.55
29	5.50	5.32	4.81	4.55
RMSE	0.94	0.93	0.80	
MAE	0.87	0.81	0.68	

**Table 7:** Probabilistic classification results.

Compound ID	CONV1D	ANN
1	[0.95, 0.05]	[0.99, 0.01]
3	[0.93, 0.07]	[0.98, 0.02]
4	[0.93, 0.07]	[0.98, 0.02]
5	[0.93, 0.07]	[0.98, 0.02]
6	[0.95, 0.05]	[0.98, 0.02]
9	[0.86, 0.14]	[0.98, 0.02]
10	[0.65, 0.35]	[0.97, 0.03]
11	[0.89, 0.11]	[0.97, 0.03]
12	[0.90, 0.10]	[0.98, 0.02]
13	[0.95, 0.05]	[0.98, 0.02]
14	[0.85, 0.15]	[0.98, 0.02]
15	[0.86, 0.14]	[0.98, 0.02]
16	[0.84, 0.16]	[0.98, 0.02]
17	[0.84, 0.16]	[0.98, 0.02]
18	[0.88, 0.12]	[0.98, 0.02]
19	[0.20, 0.80]	[0.98, 0.02]
20	[0.53, 0.47]	[0.98, 0.02]
22	[0.60, 0.40]	[0.98, 0.02]
23	[0.73, 0.27]	[0.98, 0.02]
25	[0.91, 0.09]	[0.10, 0.90]
26	[0.48, 0.52]	[0.50, 0.50]
27	[0.92, 0.08]	[0.02, 0.98]
28	[0.51, 0.49]	[0.01, 0.99]
29	[0.54, 0.46]	[0.70, 0.80]

**Table 8:** Presentation of the extra compounds added to the training procedure of the deep classification models.

Activity	Target	No. of Compounds	Training Set	Validation Set
Anti-inflammatory	COX-2	1000	850	150
Antioxidant	Nrf-2	344	292	52

## Data Availability

The data presented in this study are available on request from the corresponding author.

## Abbreviations

The following abbreviations are used in this manuscript:

LOX-3	Lipoxygenase-3
DPPH	2-Diphenyl-1-3-Picrylhydrazyl
COX-2	Cyclooxygenase-2
Nrf-2	Nuclear Factor Erythroid 2–Related Factor 2
SVR	Support Vector Regressor
ANN	Artificial Neural Network
CNN	Convolutional Neural Network
CONV1D	One Dimensional Convolutional Neural Network
ReLU	Rectified Linear Unit
RMSE	Root Mean Square Error
MAE	Mean Absolute Error
